# KLF4 transcriptionally activates non-canonical *WNT5A* to control epithelial stratification

**DOI:** 10.1038/srep26130

**Published:** 2016-05-17

**Authors:** Marie-Pier Tetreault, Daniel Weinblatt, Khvaramze Shaverdashvili, Yizeng Yang, Jonathan P. Katz

**Affiliations:** 1 Division of Gastroenterology, Department of Medicine, University of Pennsylvania Perelman School of Medicine, Philadelphia, PA USA

## Abstract

Epithelial differentiation and stratification are essential for normal homeostasis, and disruption of these processes leads to both injury and cancer. The zinc-finger transciption factor KLF4 is a key driver of epithelial differentiation, yet the mechanisms and targets by which KLF4 controls differentiation are not well understood. Here, we define *WNT5A*, a non-canonical Wnt ligand implicated in epithelial differentiation, repair, and cancer, as a direct transcriptional target that is activated by KLF4 in squamous epithelial cells. Further, we demonstrate functionally that WNT5A mediates KLF4 control of epithelial differentiation and stratification, as treatment of keratinocytes with WNT5A rescues defective epithelial stratification resulting from *KLF4* loss. Finally, we show that the small GTPase CDC42 is regulated by KLF4 in a WNT5A dependent manner. As such, we delineate a novel pathway for epithelial differentiation and stratification and define potential therapeutic targets for epithelial diseases.

Squamous epithelia provide important barriers from the outside world and are the most common sites for human cancer^1^,^2^,^3^. While some common squamous cell cancers are relatively treatable, esophageal squamous cell cancer continues to have an extremely poor prognosis, with a five-year survival of less than 20%^4^,^5^. Esophageal cancer is currently the 6^th^ most common cause of cancer death in the world, and more than 90% of these esophageal cancers are squamous cell cancers, arising within the stratified squamous epithelial cells that normally line the esophagus^3^,^6^. Moreover, esophagitis and other disorders of the esophageal lining are among the greatest sources of morbidity and healthcare costs in the United States^7^. The squamous cells that line the esophagus proliferate, differentiate, and stratify to maintain normal homeostasis and for tissue repair while providing protection against damaging luminal substances. As such, perturbation of the pathways of normal esophageal squamous epithelial differentiation contributes to both esophageal injury and cancer^8^.

Similar to the linings of the skin and several other organs, the esophageal epithelium is organized into several layers with spatial separation of cell proliferation and differentiation^9^. Esophageal epithelial proliferation occurs in the basal layer, the layer furthest from the luminal surface, and epithelial cells differentiate as they migrate upwards through the overlying suprabasal and superficial cell layers before eventually being extruded into the lumen^6^,^10^. The process of stratification involves the stacking and linking of these squamous epithelial cells during differentiation resulting in a permeability barrier. Yet, while much has been learned about the movement of these squamous epithelial cells during differentiation, the transcriptional regulation of squamous epithelial differentiation and stratification is complex^11^,^12^, and the signaling pathways that underlie squamous epithelial differentiation are not well understood.

The transcription factor *Krüppel*-like factor 4 (KLF4) is a key driver of squamous epithelial differentiation, including in the esophagus^13^,^14^,^15^,^16^. Indicative of this, KLF4 is highly expressed in differentiating esophageal epithelial cells, and genetic ablation of *Klf4* results in defective squamous epithelial differentiation. In skin, *Klf4* deletion leads to loss of barrier function and defective late-stage differentiation, with early lethality by postnatal day 1 due to these barrier defects^14^, and in the esophagus, *Klf4* ablation results in delayed differentiation, abnormal stratification, and the development of precancerous squamous cell dysplasia^16^. Moreover, KLF4 loss appears to contribute to human esophageal diseases, as *KLF4* is downregulated in human esophageal squamous cell carcinoma^17^,^18^, and *Klf4* loss also promotes skin carcinogenesis in mice^19^. Taken together, these data demonstrate a requirement of KLF4 for squamous epithelial differentiation and the relevance of KLF4 to human diseases^20^,^21^.

During squamous epithelial differentiation, *KLF4* expression is regulated by a number of factors including ZNF750, p63, and PKCδ, as well as several lncRNA^15^,^22^,^23^,^24^. These factors regulate each other as well, suggesting a coordinated network that controls squamous epithelial differentiation and converges on KLF4. Yet the mechanisms by which KLF4 controls squamous epithelial differentiation and the specific downstream targets of KLF4 during differentiation remain to be delineated. Previously, we identified the non-canonical Wnt ligand *Wnt5a*^25^ as a gene that is differentially expressed in esophageal squamous epithelial cells of mice with esophageal specific deletion of *Klf4*, compared to controls^16^, suggesting that *Wnt5a* might be a target of KLF4 in squamous epithelia. WNT5A is particularly intriguing since it is critical for differentiation as well as polarity and migration of multiple cell types^26^,^27^,^28^. In addition, WNT5A is involved in tissue repair following injury^29^ and is decreased in human esophageal esophageal squamous cell cancer^30^. In the squamous epidermis, WNT5A induces epithelial differentiation during wound healing^31^. Taken together, these findings suggest that WNT5A may be important broadly for squamous epithelial differentiation. Yet, to date, KLF4 has not been linked to non-canonical Wnt signaling, and the transcriptional control of *WNT5A* is not well-defined. Here, employing murine genetic models and primary human and mouse esophageal keratinocytes, we identify *WNT5A* as a direct transcriptional target of KLF4. Importantly, primary esophageal keratinocytes are a useful model to study epithelial differentiation, as these cells display a predominantly basal cell phenotype in culture but can be induced to terminally differentiate with high concentrations of calcium or by raising cells to the air-liquid interface, allowing the formation of fully stratified epithelia in organotypic culture^32^,^33^. Using primary keratinocytes in organotypic culture, we show that loss of *KLF4* impairs squamous epithelial differentiation and demonstrate functionally that WNT5A rescues the effects of *KLF4* loss on differentiation and stratification. Additionally, we show that KLF4 inhibits activation of the small GTPase CDC42 in a WNT5A dependent manner. As such, we define a novel mechanism for the regulation of squamous epithelial differentiation.

## Results

**Figure 1:**
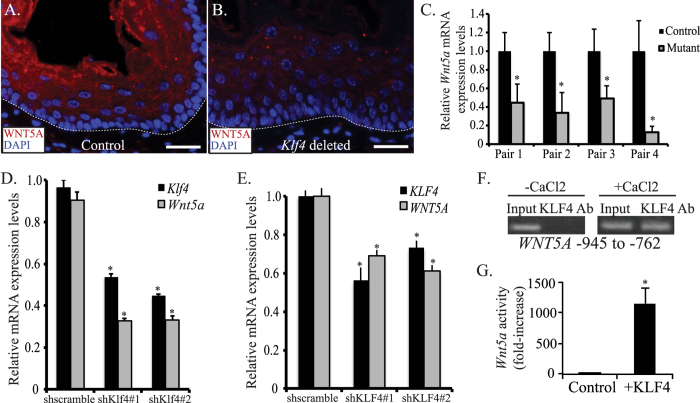
KLF4 transactivates WNT5A in esophageal epithelial cells. (**A,B**) By immunofluorescence, control mice had extensive WNT5A staining (red) in the suprabasal and superficial layers of their esophageal epithelia (**A**). In contrast, WNT5A was nearly absent from esophageal epithelia of *ED-L2/Cre;Klf4*^*loxP/loxP*^ mice (**B**). DAPI (blue) was used as a counterstain, and the white dashed line represents the approximate location of the basement membrane. Scale bars: 25 μM. (**C**) By quantitative real-time PCR, *Wnt5a* mRNA expression was decreased in the esophageal epithelium of each *ED-L2/Cre;Klf4*^*loxP/loxP*^ mouse compared to its littermate control (*p < 0.05). (**D**) *Klf4* knockdown in primary mouse esophageal keratinocytes in culture using either of two shRNA constructs resulted in a 57% decrease in *Wnt5a* mRNA levels by qPCR. (*p < 0.05) (**E**) In primary human esophageal keratinocytes, inducible *KLF4* knockdown with either of two shRNA constructs led to a 31–39% decrease in *WNT5A* mRNA expression by qPCR. (*p < 0.05) (**F**) Right panel: When human primary esophageal keratinocytes were induced to differentiate with CaCl_2_, KLF4 bound to the region of *WNT5A* between −945 to −762 upstream of the transcriptional start site. Left panel: No KLF4 binding to *WNT5A* was observed in actively proliferating keratinocytes. Lack of binding at −1992 to −1796 (not shown) confirmed specificity. (**G**) Primary mouse esophageal keratinocytes transfected with pCDNA3-Flag-Klf4 to express *Klf4* had an 1148-fold increase in luciferase reporter activity compared to cells transfected with pCDNA3.1 control. (*p < 0.05).

*ED-L2-Cre/Klf4*^*loxp/loxp*^ mice have hyperplastic esophageal epithelia with evidence of abnormal differentiation and stratification^16^. Non-canonical *Wnt5a* was reduced 3-fold on microarray studies of murine esophagus with *Klf4* deletion^16^, and we postulated that *Wnt5a* loss might be critical for the effects of *Klf4* loss. Initially, we examined the expression and localization of WNT5A in esophageal epithelial of control and *ED-L2-Cre/Klf4*^*loxp/loxp*^ mice. In esophageal epithelia of control mice, WNT5A localized to regions of cellular differentiation (Fig. 1A), a pattern of expression that overlapped with the expression domain of KLF4 (Figure S1A)^16^,^34^; WNT5A and KLF4 also co-localized in primary esophageal keratinocytes in culture (Figure S1B). In *ED-L2-Cre/Klf4*^*loxp/loxp*^ mice, WNT5A was decreased markedly within the suprabasal and superficial layers of esophageal epithelia (Fig. 1B). *Wnt5a* was also reduced at the mRNA level in esophageal epithelia of mice with *Klf4* loss compared to controls (Fig. 1C), suggesting that *Wnt5a* might be a transcriptional target of KLF4.

To mechanistically dissect the regulation of *WNT5A* by KLF4, we employed primary esophageal keratinocytes with inducible *KLF4* knockdown. *Klf4* knockdown decreased *Wnt5a* mRNA by nearly 60% in primary mouse esophageal keratinocytes (Fig. 1D), and *KLF4* knockdown decreased *WNT5A* mRNA by more than 30% in primary human esophageal keratinocytes (Fig. 1E); of note, the decreases in *Wnt5a* and *WNT5A* paralleled the reductions in *Klf4* and *KLF4*. To determine whether *WNT5A* was a direct transcriptional target for KLF4, we examined the 5′ regulatory region of human *WNT5A* for putative KLF4-binding sites, using the computational program TESS^35^ and identified a putative KLF4 binding site between −945 to −762 from the translation start site. Using ChIP assays, we demonstrated binding of KLF4 to *WNT5A* in the region of the KLF4 site (Fig. 1F). Interestingly, this binding was observed only when cells were induced to differentiate with calcium chloride (Fig. 1F, right panel). To confirm that KLF4 transactivated *Wnt5a*, we transfected primary mouse esophageal keratinocytes with a *Wnt5a* luciferase reporter^36^. Compared to control, *Klf4* transfection resulted in a 1148-fold increase in *Wnt5a* luciferase activity (Fig. 1G). Thus, KLF4 transcriptionally activates *WNT5A* during keratinocyte differentiation by binding to the 5′ regulatory region of *WNT5A*.

**Figure 2:**
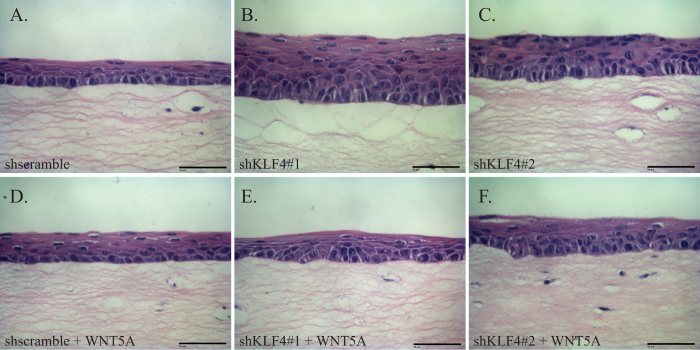
*KLF4* knockdown alters esophageal stratification via WNT5A. (**A**) In three-dimensional organotypic culture, primary human esophageal keratinocytes form stratified epithelia. Cells in the basal layer were rounded with large nuclear-cytoplasmic ratios, and cells in the suprabasal and superficial layers were flattened with compacted nuclei. (**B,C**) In contrast, *KLF4* knockdown in primary human esophageal keratinocytes in organotypic culture yielded epithelia that were hyperplastic, and cells appeared less mature, with cells outside of the basal layer maintaining a rounded appearance and large nuclei. (**D**–**F**) Treatment of the cultures with recombinant WNT5A had little effect on control epithelia (**D**) but restored normal epithelial stratification of primary esophageal keratinocytes with inducible *KLF4* knockdown (**E,F**). Scale bars, 50 μm.

To determine whether KLF4 regulates esophageal epithelial differentiation and stratification via WNT5A, we examined the effects of KLF4 and WNT5A on primary esophageal keratinocytes in three-dimensional organotypic culture. In organotypic culture, control EPC2-hTERT cells formed mature, stratified epithelia featuring rounded, proliferative cells with high nuclear-cytoplasmic ratios in the basal layer that gave rise to flattened cells with compacted nuclei in the suprabasal and superficial layers (Fig. 2A). In contrast, *KLF4* knockdown in EPC2-hTERT cells in organotypic culture yielded hyperplastic epithelia and rounded, immature-appearing cells with discernible nuclei in the suprabasal and superficial layers (Fig. 2B,C). Thus, *KLF4* knockdown in esophageal keratinocytes in organotypic culture recapitulated the esophageal phenotype of the *ED-L2-Cre/Klf4*^*loxp/loxp*^ mice (Figure S2). To test the requirement of WNT5A for KLF4 effects on esophageal epithelial stratification, we treated EPC2-hTERT cells in organotypic culture with recombinant WNT5A. Interestingly, addition of WNT5A had no effect on control EPC2-hTERT cells (Fig. 2D) while WNT5A treatment restored normal stratification of EPC2-hTERT cells with *KLF4* knockdown (Fig. 2E,F). Thus, the abnormalities of epithelial stratification resulting from *KLF4* loss are mediated by WNT5A.

**Figure 3:**
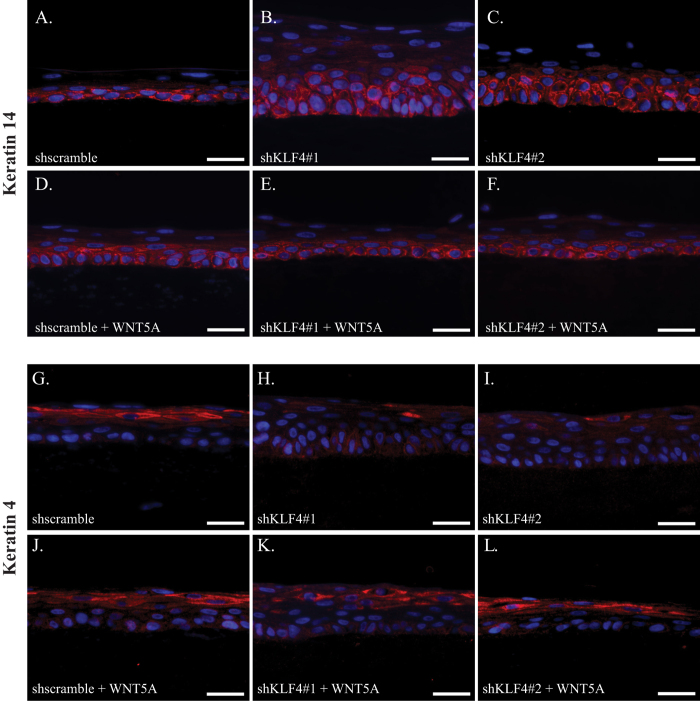
WNT5A rescues defective esophageal epithelial differentiation that results from *KLF4* loss. (**A**) Keratin 14 (red), which marks immature keratinocytes, was restricted to the basal layer in organotypic cultures of control keratinocytes. (**B,C**) When *KLF4* was knocked down in primary human esophageal keratinocytes, keratin 14 staining was more extensive, including in cells of the suprabasal layer. (**D–F**) Treatment of primary human esophageal keratinocytes with recombinant WNT5A had little effect in control cells (**D**), but restored the normal pattern of keratin 14 expression in cells with *KLF4* knockdown, with staining again restricted to the basal layer in these cells (**E,F**). (**G–I**) Keratin 4 was expressed in the suprabasal and superficial layers of organotypic cultures of control human esophageal keratinocytes (**G**) while expression was nearly absent from cells with *KLF4* knockdown (**B,C**). (**J–L**) Treatment of control primary human esophageal keratinocytes with recombinant WNT5A had minimal effect (**J**), but WNT5A treatment of organotypic cultures of cells with *KLF4* knockdown normalized keratin 4 expression, with keratin 4 staining again seen in the suprabasal and superficial layers (**K,L**). DAPI (blue) was used as a counterstain. Scale bars, 25 μm.

Normal cellular differentiation is essential for proper epithelial stratification^6^,^37^. As such, we sought to determine whether WNT5A corrected defective keratinocyte differentiation resulting from *KLF4* knockdown by examining the expression patterns of keratin 14, which marks immature, proliferative keratinocytes typically located in the basal layer, and keratin 4, a marker of keratinocyte differentiation^6^,^38^. Compared to controls (Fig. 3A), esophageal epithelia from organotypic cultures with *KLF4* knockdown had marked expansion of keratin 14 expression indicative of more immature keratinocytes (Fig. 3B,C). When control esophageal keratinocytes were treated with recombinant WNT5A (Fig. 3D), the localization of keratin 14 positive cells was similar to untreated cultures; WNT5A treatment of organotypic cultures with *KLF4* knockdown resulted in a reduction of keratin 14 expressing cells and a more normal pattern of keratin 14 expression (Fig. 3E,F). The differentiation marker keratin 4, which was expressed in suprabasal and superficial layers of control cultures (Fig. 3G), was nearly absent from organotypic cultures with *KLF4* knockdown (Fig. 3H,I), consistent with the consequences of *Klf4* loss *in vivo*^16^. Again, WNT5A showed little effect on differentiation of control keratinocytes (Fig. 3J). However, WNT5A treatment was sufficient to rescue the effects of *KLF4* knockdown on keratin 4 expression and therefore esophageal epithelial differentiation (Fig. 3K,L). Thus KLF4 controls esophageal epithelial differentiation and stratification through WNT5A.

**Figure 4:**
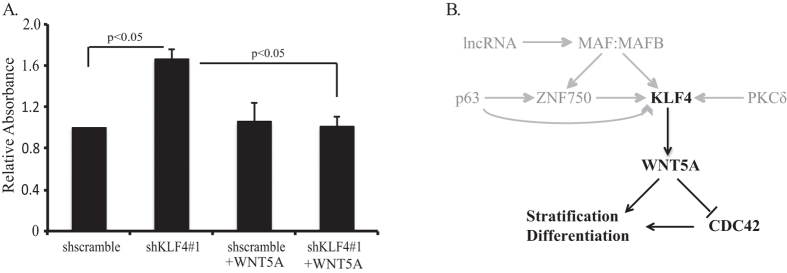
*KLF4* knockdown activates CDC42 in a WNT5A dependent manner. (**A**) Quantification of GTPase activation (n = 3) demonstrated increased CDC42 activity with *KLF4* knockdown in primary human esophageal keratinocytes induced to differentiate with CaCl_2_; activation of CDC42 in cells with *KLF4* knockdown was abolished by treatment with recombinant WNT5A. (**B**) Proposed model for the regulation of squamous epithelial cell differentiation and stratification via KLF4 and WNT5A. Previously described regulators of *KLF4* are indicated in gray.

WNT5A typically signals via the receptor tyrosine kinase ROR2 to activate β-catenin-independent Wnt pathways^39^,^40^,^41^. Members of the Rho family of GTPases, including CDC42 and RHOA, are important downstream targets of WNT5A and are critical for cellular processes such as differentiation, migration, and polarity^42^,^43^,^44^. To identify whether CDC42 and RHOA were downstream targets of KLF4-WNT5A signaling, we examined the consequences of *KLF4* knockdown on CDC42 and RHOA activation in primary human esophageal keratinocytes. Interestingly, while RHOA activation was not altered by *KLF4* knockdown (Figure S3A), *KLF4* knockdown activated CDC42, and this activation was blocked by treatment with recombinant WNT5A (Fig. 4A). Additionally, *CDC42* mRNA levels were not affected by *KLF4* knockdown (Figure S3B), indicating that KLF4 did not regulate *CDC42* transcription. Thus, in esophageal keratinocytes, KLF4 upregulates WNT5A to inhibit CDC42 activity.

## Discussion

The mucosal barrier of the esophagus is an essential line of defense against external damaging agents, and proper keratinocyte differentiation and stratification are required to maintain the integrity of the epithelial barrier^6^,^8^. The *Krüppel*-like factor family member KLF4 is critical for the regulation of epithelial homeostasis and disease, including in the squamous esophagus and skin, and mice with *Klf4* deletion in esophageal keratinocytes develop altered cell morphology, delayed differentiation, and abnormal stratification, leading to precancerous esophageal squamous cell dysplasia^14^,^16^,^20^. Yet, the molecular mechanisms by which KLF4 controls squamous epithelial differentiation and stratification have not been clear.

Non-canonical WNT5A signaling has been extensively studied in development where it regulates cell polarity and directional cell movement, but the importance of WNT5A for adult epithelial stratification is less clear^43^. Recently, WNT5A was shown to restore stratification of the apical ectodermal ridge in the developing limb^45^. *Wnt5a* deletion in mice compromises differentiation of the hair follicle^36^, and in the interfollicular epidermis, WNT5A activation induces keratinocyte differentiation during wound healing^31^, consistent with a role for WNT5A in tissue repair seen in other contexts^29^. Our findings here highlight the importance of non-canonical WNT5A for epithelial squamous differentiation and stratification, and since WNT5A treatment of normal epithelia has no overt effects on differentiation and stratification, WNT5A might be effective to therapeutically target defective differentiation and/or stratification and to promote esophageal wound healing following injury. Moreover, *Wnt5a* is decreased in a murine model of esophageal squamous cell carcinogenesis^46^ suggesting that WNT5A may have tumor suppressive function in esophageal squamous cell carcinogenesis. Nonetheless, while organotypic cultures are useful models of carcinogenesis^32^,^47^,^48^,^49^, overexpression of *Klf4* results in esophageal squamous cell cancer via activation of inflammatory pathways *in vivo*^50^ and thus further study is required to exclude that higher levels of WNT5A, which is downstream of KLF4, promote esophageal inflammation and carcinogenesis *in vivo*.

In squamous epithelia, KLF4 is regulated directly and/or indirectly by the transcription factors p63, ZNF750, MAF, and MAFB, by PKCδ, and by the lncRNA ANCR and TINCR^15^,^22^,^23^,^24^. Integrating our data with the published literature, we propose a broad network that controls squamous epithelial differentiation and stratification, converging on KLF4 and WNT5A (Fig. 4B). Interestingly, CDC42 is inhibited by WNT5A in esophageal keratinocytes, while WNT5A may activate CDC42 or the WNT5A and CDC42 pathways may cooperate in other contexts^51^,^52^,^53^. CDC42 itself can either promote or inhibit differentiation^54^,^55^, and in fact, both upregulation and downregulation of CDC42 activity can inhibit cell growth in the same cell type, suggesting that tight regulation of CDC42 may be essential for normal differentiation^54^,^56^. In addition, both WNT5A and CDC42 can regulate β-catenin-dependent Wnt signaling, which may play a role in esophageal squamous cell carcinogenesis^57^,^58^,^59^,^60^. In the stomach, WNT5A from gastric innate lymphoid cells activates epithelial RHOA, in contrast to our findings, suggesting that the pathways downstream of WNT5A vary by context^61^. Thus the contextual and coordinate functions of KLF4, WNT5A, and CDC42 in epithelial differentiation, stratification, and carcinogenesis require further study.

In sum, we delineate a novel pathway for epithelial differentiation and stratification acting via the key differentiation-promoting transcription factor KLF4 and the non-canonical Wnt ligand WNT5A. A number of key differentiation factors converge on KLF4^15^,^22^,^23^,^24^, and we demonstrate that loss of *KLF4* leads to defects in epithelial differentiation and stratification that are rescued by WNT5A. Thus, in outlining the mechanisms underlying squamous epithelial differentiation and stratification, we define potential therapeutic targets for diseases and disorders of the esophagus and other stratified squamous epithelia, sources of significant human morbidity and mortality^1^,^2^,^3^,^7^. Moreover, as KLF4 is important for cellular differentiation and carcinogenesis more broadly^20^,^21^, these targets may also be relevant to other tissues and cell types.

## Methods

### ED-L2-Cre/Klf4^loxP/loxP^ mice

All animal studies were approved by the Institutional Animal Care and Use Committee (IACUC) at the University of Pennsylvania and carried out in accordance with the approved guidelines. Mice homozygous for the floxed *Klf4* gene and hemizygous for the *ED-L2/Cre* transgene have been previously described^16^. For analyses, esophagi from 3 month-old mice were removed and processed as described^16^. For experiments with *ED-L2*-Cre/*Klf4*^*loxP/loxP*^ mice, sex-matched littermate *Klf4*^*loxP/loxP*^ mice lacking the *Cre* transgene served as controls. All mice used for experiments were on a mixed genetic background.

### Cell Culture and Treatment

The isolation and culture of primary mouse esophageal keratinocytes were described elsewhere^62^. Primary human esophageal keratinocytes (EPC2) retrovirally transduced with *hTERT* to generate EPC2-hTERT cells^63^ were cultured as previously described^47^. HEK 293T cells used for lentivirus production were purchased from ATCC. For WNT5A treatment, recombinant mouse/human WNT5A (R&D Systems) was added at 100 ng/ml into growth media.

### Viral constructs and infections

The lentiviral vector pLKO.1 puro^64^, a gift from Bob Weinberg (Addgene plasmid # 8453) was used to express 2 distinct short hairpin RNAs (shRNA) against mouse *Klf4*, and the lentiviral vector TET-pLKO-neo^65^, a gift from Dmitri Wiederschain (Addgene plasmid # 21916), was used to express 2 distinct shRNA against human *KLF4*. For TET-pLKO-neo, shRNA was induced in cells in two-dimensional culture with 4 μg/ml doxycycline for 7 days. Additional methods are available in Supplemental Information.

### Immunohistochemistry/Immunofluorescence/Western Blotting

Immunohistochemistry, immunofluorescence, and Western blots were performed using standard protocols. For descriptions of the protocols and antibodies used, see the Supplemental Information.

### RNA analyses

RNA was extracted from primary esophageal keratinocytes using the GeneJet RNA Purification Kit (Thermo Fisher Scientific) following the manufacturer’s instructions. Reverse transcription was performed with the Maxima First-Strand cDNA Synthesis kit (Thermo Fisher Scientific). Quantitative real-time PCR (qPCR) was performed in triplicate using an ABI Step-One Plus sequence detection system (Thermo Fisher Scientific) and SYBR Green PCR master mix (Thermo Fisher Scientific). TATA box binding protein gene (TBP) and GAPDH were used as internal controls. Primer sequences are available in Supplemental Information.

### Chromatin Immunoprecipitation (ChIP) Assay

ChIP assays were performed in triplicate with the ChIP assay kit (Millipore) as described previously^47^. Cells were treated with 1% formaldehyde for 10 minutes to cross-link associated protein to DNA, lysed, and sonicated. After a 10-fold dilution, samples were pre-cleared with protein A-agarose/salmon sperm DNA for 30 minutes at 4 °C and incubated overnight at 4 °C with 1:500 anti-KLF4 antibody^66^ or 1:500 anti-mouse IgG (Sigma) as a negative control. Additional methods are available in Supplemental Information.

### Reporter assays

The Wnt5a-luc reporter plasmid, containing the region from −1.66 to + 2.29 kb relative to the mouse *Wnt5a* transcription start site in the pGL4 Luciferase Reporter Vector (Promega), was a gift of G. Paolo Dotto^36^. To express *Klf4*, a Flag-tagged full-length mouse *Klf4* cDNA was subcloned into the pCDNA3.1 vector (Life Technologies). Mouse primary esophageal keratinocytes were transfected with either pCDNA3.1 or pCDNA3-Flag-Klf4 and with either pGL4 or Wnt5a-luc at 70% confluence in triplicate on 24-well plates using Turbofect transfection reagent (Thermo Fisher Scientific). Cells were lysed after 48 hours with Cell Lysis Buffer (Pharmingen), and luciferase reporter activity was analyzed using luciferase assay reagent (Promega) with a GLOMAX multi detection system (Promega). Luciferase activity was normalized to Renilla and expressed as relative luciferase activity.

### Organotypic culture

EPC2-*hTERT* cells containing TET-pLKO-neo constructs were grown in three-dimensional organotypic culture as described previously^32^. *KLF4* knockdown was induced with 4 μg/ml of doxycycline from days 7–15, and recombinant WNT5A was added from days 11–15. Cultures were fixed overnight in 10% buffered formalin phosphate (Fisher Scientific) before paraffin embedding and sectioning.

### CDC42 and RHOA activation assays

G-LISA CDC42 and RHOA activation assays (Cytoskeleton Inc.) were performed according to the manufacturer’s instructions. Briefly, 50 μg of protein lysates were incubated at 4 °C for 30 minutes under agitation. After washes and incubation with the antigen presenting buffer, the plates were incubated with primary and secondary antibodies at room temperature for 45 minutes. Absorbance at 490 nm was measured using a Tecan Infinite 200 PRO microplate reader (Tecan) following a 15 minute incubation with the HRP detection reagent.

## Additional Information

**How to cite this article**: Tetreault, M.-P. *et al*. KLF4 transcriptionally activates non-canonical *WNT5A* to control epithelial stratification. *Sci. Rep*. **6**, 26130; doi: 10.1038/srep26130 (2016).

## Supplementary Material

Supplementary Information
