# Supplementary material for: KLF4 transcriptionally activates non-canonical WNT5A to control epithelial stratification
Source: Sci Rep. 2016 May 17;6:26130. doi: 10.1038/srep26130 (PMC4869036; doi:10.1038/srep26130)
Supplement: Supplementary Information [file srep26130-s1.pdf]

Title: KLF4 transcriptionally activates non-canonical *WNT5A* to control epithelial stratification

Authors: Marie-Pier Tetreault[1, 2], Daniel Weinblatt[1], Khvaramze Shaverdashvili[1], Yizeng Yang[1], and Jonathan P. Katz[1,*]

Affiliations: [1]Division of Gastroenterology, Department of Medicine, University of Pennsylvania Perelman School of Medicine, Philadelphia, PA USA

[2]Current address: Department of Medicine, Gastroenterology and Hepatology Division, Northwestern University Feinberg School of Medicine, Chicago, IL 60611 USA

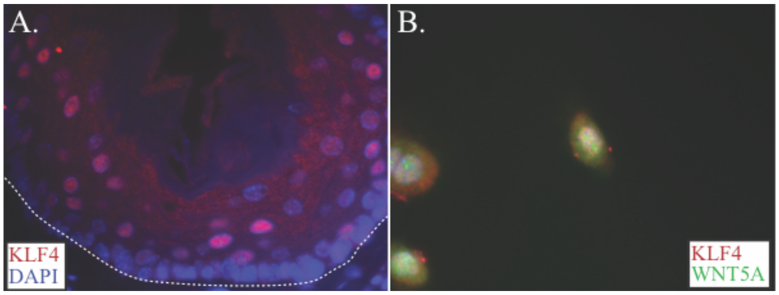

Figure S1. KLF4 co-localizes with WNT5A in esophageal epithelial cells. (A) By immunofluorescence, KLF4 staining (red) localized predominantly to the suprabasal and superficial layers of murine esophageal epithelia, the same pattern of expression as for WNT5A in Figure 1A. DAPI (blue) was used as a counterstain. The white dashed line represents the approximate location of the basement membrane. (B) KLF4 (red) and WNT5A (green) co-localized in primary esophageal keratinocytes in culture.

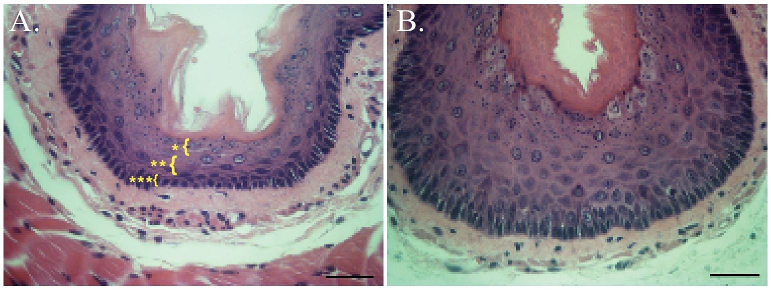

Figure S2. Loss of *Klf4* alters esophageal epithelial differentiation and stratification *in vivo*. (A-B) While esophageal squamous epithelial cells from control mice differentiated and stratified normally (A), esophageal epithelia from *ED-L2/Cre;Klf4*$^{loxP/loxP}$ mice were hyperplastic, and keratinocytes in the suprabasal and superficial layers appeared rounded and immature, indicative of perturbed squamous differentiation pathways (B). The individual layers of the normal esophageal epithelium are indicated in (A): *, superficial layer; **, suprabasal layer; and ***, basal layer. Scale bars: 25µM.

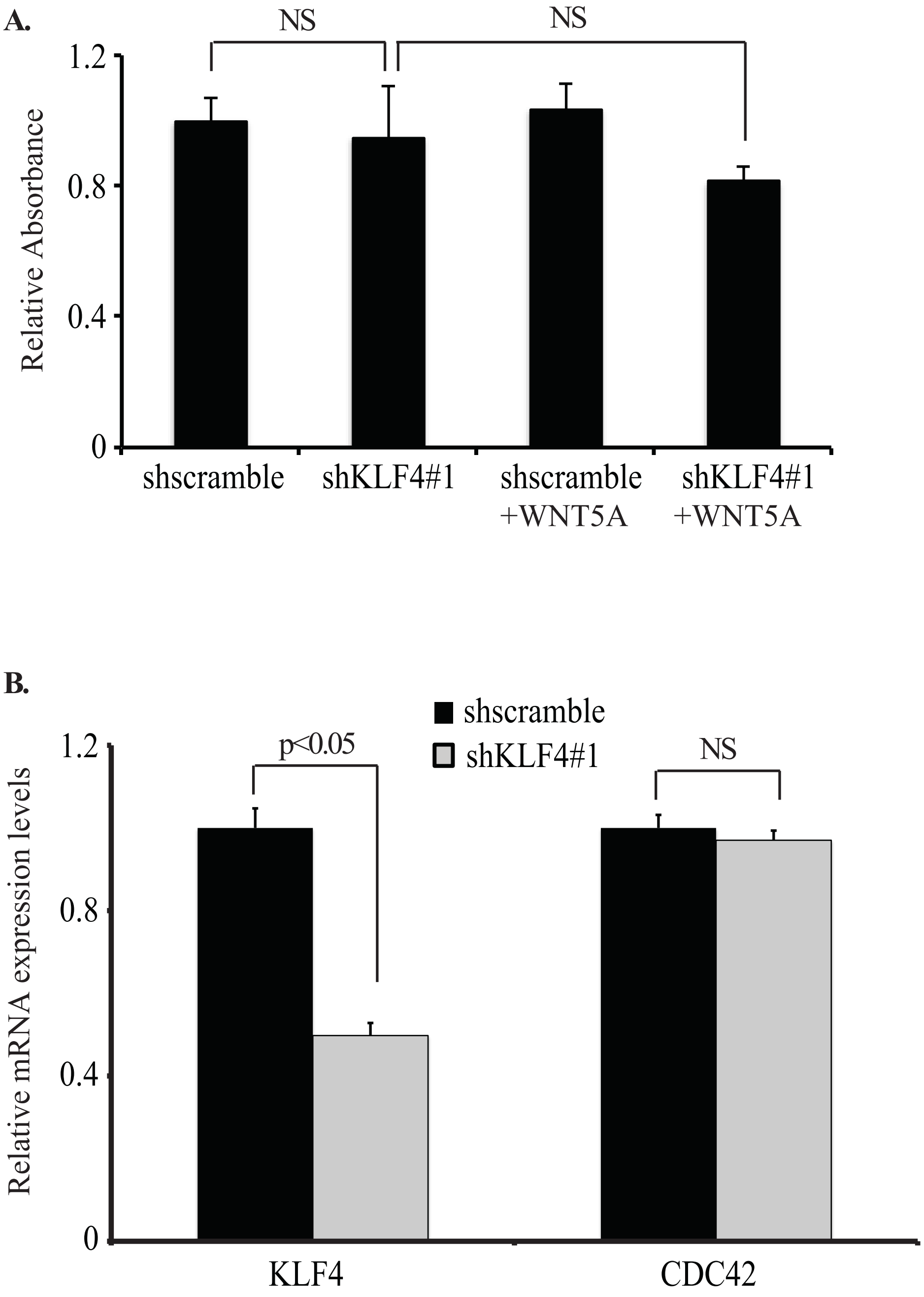

Figure S3. KLF4 does not regulate RHOA activation or *CDC42* mRNA expression. (A) Quantification of GTPase activation revealed no change in RHOA activity in primary human esophageal keratinocytes with *KLF4* knockdown or with recombinant WNT5A treatment. (B) *KLF4* knockdown in primary human esophageal keratinocytes did not alter *CDC42* mRNA levels by qPCR. (NS, not significant)

Supplementary Methods

Viral constructs and infections

Lentivirus was packaged by transfecting into HEK 293T cells with the packaging plasmids pMD2.G (Addgene plasmid #12259) and psPAX2 (Addgene plasmid #12260), which were gifts from Didier Trono. Virus-containing medium was harvested 48 hours and 72 hours after transfection and filtered with 0.45µM Millex HV filters (EMD Millipore). Keratinocytes were then infected with culture supernatants from individual HEK 293T cells at a 1:20 dilution in KSFM and selected after 48 hours with 150 µg/ml G418 (Life Technologies) or 1.5µg/ml puromycin (Sigma) for 10 days.

Western blotting

For each sample, 20 µg of total protein were separated on a NuPAGE 4–12% bis-tris acrylamide gel (Life Technologies) and transferred onto polyvinylidene difluoride membrane (EMD Millipore, Temecula, CA). After blocking with 5% non-fat milk in PBS with 0.2% Tween-20, membranes were incubated overnight at 4 °C with 1:10,000 rabbit anti-KLF4, which we generated previously[1], or 1:1000 rabbit anti-human ROR2 (Cell Signaling); membranes were then incubated with a 1:10,000 dilution of anti-rabbit/horseradish peroxidase or anti-mouse/horseradish peroxidase (GE Healthcare Life Sciences) or goat/horseradish peroxidase (Santa Cruz Biotechecnology Inc.) and developed with the enhanced chemiluminescence plus Western blot analysis kit (EMD Millipore).

Immunofluorescence/Immunohistochemistry

For immunohistochemistry and immunofluorescence on tissue sections, we performed antigen retrieval (Tris-Base pH 8.0 for WNT5A; Citric Acid Buffer pH 6.0 for all other antibodies) and incubated with one of the following antibodies: WNT5A (R&D Systems, 1:50 or 1:100), KLF4 (1:2,500)[1], Cytokeratin 4 (Thermo Fisher Scientific, 1:250), Cytokeratin 14 (Thermo Fisher Scientific, 1:150). Species-specific secondary antibodies were added (CyTm3 AffiniPure antibodies, Jackson Immunoresearch), and antibody binding was detected. Images were captured on a Nikon Eclipse E600 microscope and Photometrics CoolSNAP CCD camera (Roper Scientific). Immunofluorescence of cells was performed on Millicell EZ Slides (EMD Millipore). Cells were fixed with neutral buffered formalin for 10 minutes, incubated with antibodies as outlined above, and images were captured on a Nikon Eclipse Ti-U microscope attached to a Yokagawa CSU-10 confocal scanner unit.

ChIP assays

Cells were precipitated with protein A-agarose for 1 hour, heated at 65 °C for 4 hours, treated with proteinase K, and DNA-extracted with phenol/chloroform. Primers were designed to amplify the region from -945 to -762 and from -1992 to -1796 of the 5′ regulatory region of the human *WNT5A* gene as below:

**Region of *WNT5A***	**Forward Primer (5' to 3')**	**Reverse Primer (5' to 3')**
-945 to -762	AGCAAAGGCAATTGGAGAGA	GAGTCCAAGGCAGTTCGTGT
-1992 to -1796	TGCAAGGGGAAACATGAAGT	CCCTTCAATAGTGGGTAGGG

PCR was performed with puReTaq Ready-to-Go PCR beads (Amersham Biosciences) for 25 cycles at 95 °C for 1 min, 55 °C for 1 min, and 72 °C for 1 min. PCR products were separated on a 2% agarose gel and visualized by a Gel Doc XR+ system (Bio-Rad).

Primer sequences for quantitative real-time PCR

Primer sequences for quantitative real-time PCR are detailed below:

**Gene**	**Forward Primer (5' to 3')**	**Reverse Primer (5' to 3')**
Mouse *Klf4*	GTGCCCCGACTAACCGTTG	GTCGTTGAACTCCTCGGTCT
Mouse *Wnt5a*	GATTGTCCCCCAAGGCTTAAC	CACTCCCGGGCTTAATATTCC
Mouse *Tbp*	CAACAGCCTTCCAACTTATGC	TGGAGTAAGTCCTGTGCCGTA
Human *KLF4*	CCCCGTGTGTTTACGGTAGT	GCGGCAAAACCTACACAAAG
Human *WNT5A*	GTATCAGGACCACATGCAGT ACATC	GGAATTGATACTGGCATTCTTTGA
Human *TBP*	TGTACCGCAGCTGCAAAAT	GGATTATATTCAGCGTTTCG
Human *GAPDH*	GAAGGTGAAGGTCGGAGTCA	AATGAAGGGGTCATTGATGG

Supplementary References

1 Yang, Y., Goldstein, B. G., Chao, H. H. & Katz, J. P. KLF4 and KLF5 regulate proliferation, apoptosis and invasion in esophageal cancer cells. *Cancer Biol Ther* **4**, 1216-1221 (2005).
